# Vertex-element models for anisotropic growth of elongated plant organs

**DOI:** 10.3389/fpls.2013.00233

**Published:** 2013-07-10

**Authors:** John A. Fozard, Mikaël Lucas, John R. King, Oliver E. Jensen

**Affiliations:** ^1^Agricultural and Environmental Sciences, Centre for Plant Integrative Biology, School of Biosciences, University of NottinghamLeics, UK; ^2^Institut de Recherche pour le Développement, UMR DIADEMontpellier, France; ^3^School of Mathematical Sciences, University of NottinghamNottingham, UK; ^4^School of Mathematics, University of ManchesterManchester, UK

**Keywords:** multiscale, simulation, microfibrils, viscosity, anisotropy

## Abstract

New tools are required to address the challenge of relating plant hormone levels, hormone responses, wall biochemistry and wall mechanical properties to organ-scale growth. Current vertex-based models (applied in other contexts) can be unsuitable for simulating the growth of elongated organs such as roots because of the large aspect ratio of the cells, and these models fail to capture the mechanical properties of cell walls in sufficient detail. We describe a vertex-element model which resolves individual cells and includes anisotropic non-linear viscoelastic mechanical properties of cell walls and cell division whilst still being computationally efficient. We show that detailed consideration of the cell walls in the plane of a 2D simulation is necessary when cells have large aspect ratio, such as those in the root elongation zone of *Arabidopsis thaliana*, in order to avoid anomalous transverse swelling. We explore how differences in the mechanical properties of cells across an organ can result in bending and how cellulose microfibril orientation affects macroscale growth. We also demonstrate that the model can be used to simulate growth on realistic geometries, for example that of the primary root apex, using moderate computational resources. The model shows how macroscopic root shape can be sensitive to fine-scale cellular geometries.

## 1. Introduction

Through advances in molecular biology, much information has been gathered about plant hormone response networks (Santner et al., 2009) and the mathematical modeling of small regulatory networks has led to increased understanding of their function (Middleton et al., 2012a). New reporters for hormone levels (Band et al., 2012b; Brunoud et al., 2012), combined with image analysis tools (Pound et al., 2012), now permit the acquisition of data for individual cells within a whole organ. However, a current challenge is to understand how cell-scale processes influence behavior at organ scales; in particular, how variations in the mechanical properties of individual plant cell walls, affected by cell-wall-remodeling enzymes under the control of various hormones and regulatory networks, in turn control growth on the whole-organ scale. The complexity of this process, involving hydraulics, turgor pressure regulation, and a large number of biochemical changes in the constituents of the cell wall, necessitates a modeling approach in which these factors can be integrated. Multicellular models which incorporate cell mechanical properties are necessary to understand how the growth of individual cells is organized to drive organ-scale growth and development (Band et al., 2012a). For instance, spatially one-dimensional models which treat the root as a line of cells have been used to examine the regulation of growth by hormones (Chavarría-Krauser and Schurr, 2004; Chavarría-Krauser, 2006; Muraro et al., 2013).

Vertex-based models (Weliky and Oster, 1990; Nagai and Honda, 2001) are an attractive framework for examining the effects of cell-scale processes on behavior at the whole-organ scale, as they explicitly treat each cell as an individual object. Symplastic growth (Priestley, 1930), where neighboring cell walls adhere and do not slide across each other, can be imposed automatically by the sharing of vertices and walls between adjacent cells, and many of the complications in simulations of animal tissues (caused by cells separating and sliding over each other) are considerably less significant for plant organs. However, standard implementations of vertex-based models can be inadequate to represent elongating organs such as roots because of the large aspect ratio of cells, which makes it difficult to prevent the transverse swelling of cells (normal to the primary axis of the organ).

Simple mass-spring-based models [e.g., vertex-vertex systems (Smith et al., 2003)] and vertex-based models (Rudge and Haseloff, 2005; Dupuy et al., 2006; Jönsson et al., 2006; Smith et al., 2006; Dupuy et al., 2008; Hamant et al., 2008; Merks et al., 2011; Abera et al., 2013; Alim et al., 2012; Uyttewaal et al., 2012) have previously been used to examine plant organ growth. These models represent three-dimensional (3D) plant tissues as a collection of 2D polygons [possibly on a 3D surface, e.g., in Smith et al. (2006) and Hamant et al. (2008)] in order to simplify computation. They capture mechanical processes such as cellulose microfibril reorientation and anisotropic viscous properties of the cell walls to only a limited extent, as they do not explicitly consider cell walls in the plane of the 2D simulation. None of these models has been applied to a growing primary root, for example. More sophisticated finite-element models for plant organs exist (Jönsson et al., 2006; Huang et al., 2012), but implementing cell division within these models, whilst possible, is a complex task. To address these problems, we present here a new 2D “vertex-element” model, combining vertex-based simulations with a finite-element discretization of in-plane walls, which is computationally efficient and capable of simulating problems on the scale of a whole organ, which in the present case is the elongating section of the root of *Arabidopis thaliana*. This method has similarities to the cell-level finite-element methods for animal cells developed by the Brodland group (Chen and Brodland, 2000; Brodland et al., 2007), but is tailored to the distinct mechanical properties of plant cells.

The composite structure of the primary plant cell wall endows it with complex properties that can be independently regulated by a variety of enzymatic and metabolic processes (Geitmann, 2010). Oriented cellulose microfibrils promote anisotropic elongation in the direction orthogonal to the fibrils (Baskin, 2005). The microfibrils are embedded in a pectin matrix and are crosslinked by xyloglucan polymers (Cosgrove and Jarvis, 2012). A variety of constitutive models have been proposed for the different wall components at different levels of organization. At the level of a whole cell or tissue, elongation is commonly modeled as a viscoplastic process, parameterized by a yield stress and an extensibility (Lockhart, 1965). Continuum models at the level of the wall have demonstrated how fiber reorientation (in addition to matrix stiffening) can suppress cell elongation (Dyson and Jensen, 2010) and have shown how crosslinks and matrix properties might independently contribute to yield and extensibility properties (Dyson et al., 2012). Macromolecular simulations (Kha et al., 2010; Yi and Puri, 2012) give insights into how detailed aspects of chemical structure influence mechanical properties. This hierarchy of complementary modeling approaches must be integrated in order to understand plant tissue growth fully. Here, we lay out a computational framework for connecting properties of individual cell walls to a multicellular tissue (i.e., spanning scales from cell walls to the organ level), using the Arabidopsis root as a template.

To illustrate the potential of our approach, to understand basic properties of the multicellular simulation tool (particularly its capacity to characterize the biomechanics of a growing tissue) and to discuss some of the potential pitfalls in its computational implementation, we show how differences in mechanical properties between cell files in an elongated organ can affect growth at the organ level. In particular, we increase the extensibility of a single cell file in a simple organ geometry, causing the growing organ to bend. We also explore the retardation of cell elongation though reorientation of microfibrils. Then, to show how mechanical properties can be readily controlled by hormone levels in such a framework, we consider a simple model in which cell mechanical properties (specifically, yield stress) are regulated by a substance (a growth inhibitor) which is produced in the root apex and undergoes passive diffusion between cell compartments. Cell division is also included in this simulation. The geometry used to initialize the simulation is based upon confocal imaging. We use the model to illustrate the impact of detailed cellular geometries on organ-scale morphology.

## 2. Materials and methods

### 2.1. Vertex-element model

**Figure 1:**
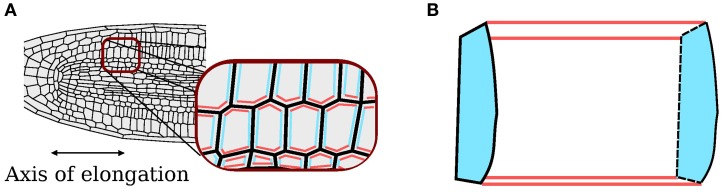
**Axial and cross walls. (A)** Classification of cell walls perpendicular to the plane of the simulation as axial (red) and cross (blue); walls in the plane of the simulation are shaded gray. **(B)** Corresponding walls in the 3D organ.

Simulations are performed in a 2D plane; for the current problem this is a 2D longitudinal section through the midline of the root, as illustrated in Figure 1. The 2D model captures aspects of the 3D structure by distinguishing three classes of wall: those in the plane of the simulation; those perpendicular to the plane of the simulation and aligned with the direction of elongation (“axial” walls); and those perpendicular to the plane of the simulation and orthogonal to the direction of elongation (“cross” walls). Cross walls represent the approximately circular end plates of elongating cells, whilst the remaining wall types represent the curved walls parallel to the direction of elongation. In the 2D representation, cells occupy polygonal regions, the boundaries of which are described by a list of edges, each edge being a line joining two vertices. Adjacent cells share edges. Whilst the common edge is treated as a single entity for the representation of the organ geometry, for mechanical purposes we consider the physical cell walls associated with each of the two cells separately. This structure automatically ensures that the organ remains contiguous, as is the case during symplastic growth.

The organ grows primarily through the motion of its vertices. Other changes, such as refining an edge into two smaller edges or the introduction of new walls during cell division, may (where necessary) be performed during the simulation. Topological transitions, which are important in animal epithelial tissues and soap bubble rafts, do not occur here because of the inability of neighboring plant cell walls to slide across each other or of an edge section to shrink to zero length, except during a small number of special events such as lateral root emergence, abscission, cell death etc.

#### 2.1.1. Forces

Mechanical simulation using such a model involves consideration of the forces acting on each of the vertices. These are viscous and elastic forces from walls perpendicular and parallel to the plane of the simulation, and pressure forces acting on the boundaries of each cell. Owing to the small length scale of the cells, inertia is taken to be negligible, so these forces are always in balance. The forces acting on each vertex are calculated as functions both of the vertex positions and of the velocities, and equated to zero. This gives a system of differential equations relating the vertex positions and velocities, which are integrated numerically. The specific forms of the forces used are described in more detail below; in order for the system to be numerically well-behaved (more precisely, to be a system of differential, rather than differential-algebraic, equations) and to avoid transverse cell expansion, the mechanical properties of cell walls perpendicular to the plane of the simulation are considered. In order to prevent solid-body translation and rotation of the growing organ, the positions of vertices on the shootward (here corresponding to the right-hand) end of the organ are fixed.

***2.1.1.1. Turgor pressure***. Plant cells maintain their shape though an internal turgor pressure, which is generated by osmosis. Turgor pressure is taken to be constant and uniform in all cells; whilst experimental measurements (Pritchard et al., 1993) and theoretical considerations (Chavarría-Krauser et al., 2005) indicate that the turgor pressure is likely to decrease along the axis, in both cases this change was relatively small (<10%). There is some evidence that it varies between cell types (and that for roots it is higher in cortical than epidermal cells) (Javot et al., 2003; Passioura and Boyer, 2003).

**Figure 2:**
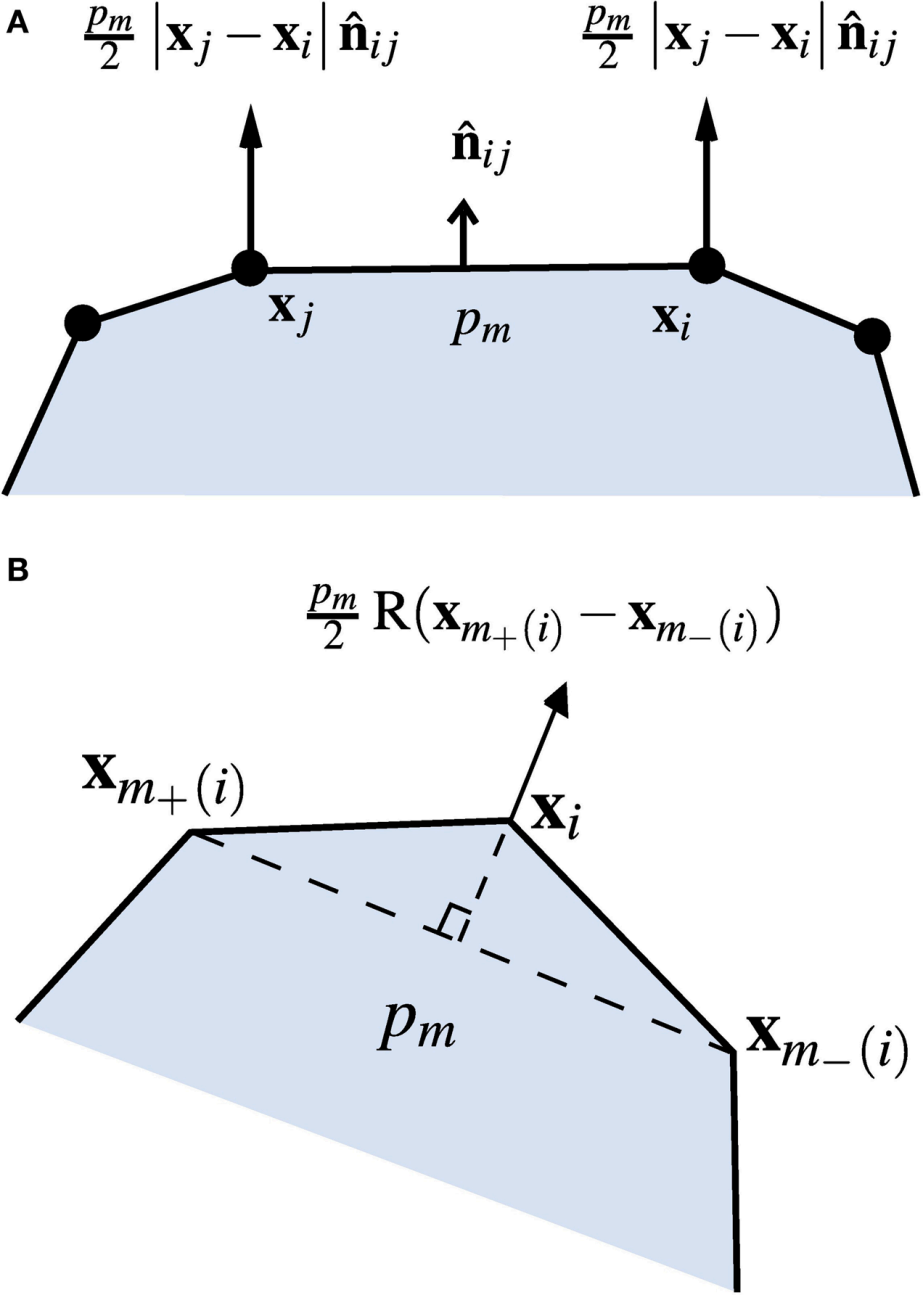
**Turgor pressure forces. (A)** Forces from the pressure *p*_*m*_ in cell *m* acting on the wall between vertices **x**_*i*_ and **x**_*j*_. **(B)** Total force acting on the vertex **x**_*i*_ from the pressure in cell *m*.

This pressure exerts an outwards force on each cell edge (per unit length normal to the plane of the simulation), normal to the edge and with magnitude proportional to its length, as shown in Figure 2A. Consider the edge *e* between the vertices with indices *i* and *j*, with positions **x**_*i*_ = (*x*_*i*_, *y*_*i*_) and **x**_*j*_ = (*x*_*j*_, *y*_*j*_). If these describe a wall of the cell with index *m* (and internal pressure *p*_*m*_) in the positive (anti-clockwise) direction, the force on the wall due to the pressure in cell *m* is

(1) $$p_{m}|x_{j}-x_{i}|{\hat{n}}_{ij}\equiv p_{m}\text{R}(x_{j}-x_{i}),\;{\hat{n}}_{ij}\equiv \frac{\left(y_{j}-y_{i},x_{i}-x_{j}\right)}{\left|x_{j}-x_{i}\right|}.$$

Here R(**x**) ≡ **x** × **k**, where **k** is the unit vector pointing out of the plane, corresponding to in-plane rotation by −π/2, and × denotes the vector product. The turgor force is taken to be equally distributed between the two vertices, giving the contributions

(2) $$\frac{p_{m}}{2}\text{R}(x_{j}-x_{i})$$

to the total forces acting at **x**_*i*_ and **x**_*j*_. The total pressure force on each vertex is the sum of the contributions from all the cells of which it is on the boundary, i.e., the force on vertex *i* can be expressed in the form

(3) $$\sum_{m|i\;\in B(m)}\frac{p_{m}}{2}\text{R}\left(x_{m_{+}(i)}-x_{m_{-}(i)}\right)$$

where the sum runs over all cells *m* for which the vertex with index *i* is within its set of boundary vertices *B*(*m*), and *m*_−_(*i*) and *m*_+_(*i*) denote the indices of the previous and next vertices to *i* around cell *m* in an anticlockwise direction (see Figure 2B).

***2.1.1.2. Constitutive assumptions***. Turgor pressure forces are resisted by the cell walls of the tissue, which are assumed here to be under tension. The cell walls have a strongly anisotropic distribution of cellulose microfibrils embedded in a viscous matrix; these microfibrils increase the resistance to deformation of the cell wall in the fiber directions, thereby promoting elongation perpendicular to these directions. In simulating growth, over timescales of hours, axial cell elongation is determined by irreversible creep of the axial cell walls, which can be modeled using a viscoplastic constitutive law (Passioura and Fry, 1992; Cosgrove, 1993).

Wall stresses in the current configuration are obtained from a non-linear anisotropic constitutive law for the Cauchy stress resultant **σ**, which we assume takes the form

(4) $$\sigma =\sigma_{y}+\sigma_{a}+\sigma_{e}.$$

Here **σ**_*y*_ is a non-linear isotropic viscous contribution (incorporating yielding effects), **σ**_*a*_ is an additional anisotropic viscous term which includes the effects of the cellulose microfibrils and **σ**_*e*_ is the elastic contribution to the stress tensor. We attribute different combinations of these components to the three classes of walls in the simulation. We also assume that walls are sufficiently thin for their bending resistance to be negligible. We do not take explicit account of wall thickness but instead lump such factors into material parameters appearing below.

We model the non-linear isotropic component by

(5) $$\sigma_{y}=2\left(\mu_{1}+\frac{\tau_{w}}{\varepsilon^{*}}\left(1-\exp\left(-\frac{{\text{ε}}^{*}}{\varepsilon }\right)\right)\right)Ε,$$

where

(6) $$\varepsilon \equiv \sqrt{\sum_{i=1}^{2}\sum_{j=1}^{2}E_{ij}^{2}}.$$

Here Ε is the rate-of-strain tensor, μ_1_ is an isotropic viscosity associated with the pectin matrix (plus embedded hemicellulose crosslinks), and τ_*w*_ and ε^*^ are the yield stress and yield strain rate of the wall. This expression is adapted from the (one-dimensional) model of Dyson et al. (2012), and has the advantage of being related directly to the properties of cross-links between cellulose microfibrils. If we assume τ_*w*_/ε^*^ » μ_1_, the wall is then more extensible in the post-yielded state (ε » ε^*^) and stiffer in the pre-yielded state (ε « ε^*^). This non-linear model is similar to those used to regularize numerical simulations of visco-plastic (Bingham) fluids (Papanastasiou, 1987) and generalizes the traditional Lockhart equation (Lockhart, 1965). For simplicity, as in Dyson and Jensen (2010), we assume that the cell wall remains of constant thickness (through the addition of new material to the cell wall) in the current configuration even as the wall is stretched. For simulations on a synthetic geometry (Figures 5–7 below), we set τ_*w*_ = 0.

We include the effect of microfibrils by considering a pair of microfibril directions, **a**_1_ and **a**_2_, that are embedded in the material of the cell wall and are advected with it. The resulting anisotropic viscous stresses are given by

(7) $$\sigma_{a}=\sum_{k=\;1}^{2}\left\{\mu_{2}(a_{k}\cdot \;Εa_{k})a_{k}⊗a_{k}+\mu_{3}(a_{k}⊗\;Εa_{k}+\;Εa_{k}⊗a_{k})\right\}$$

where the symbol ⊗ denotes the outer product of two vectors (**a** ⊗ **b** = **a b**^*T*^). The viscosity μ_2_ represents the additional resistance to extension in the fiber directions; **a**_*k*_ · Ε**a**_*k*_ gives the strain rate in the direction parallel to the fibers and **a**_*k*_ ⊗ **a**_*k*_ is a tensor which represents the direction of the fiber family. μ_3_ is an additional viscosity which resists shear of the cell wall parallel to the fiber direction. When μ_2_ is chosen to be much larger than μ_1_, the cell wall element has low extensibility in the direction of the fibers, and this leads to anisotropic cell expansion (Dyson and Jensen, 2010). We note that (Equation 7) is readily extended to include a distribution of fiber angles; for simplicity we restrict attention here to just two primary fiber directions.

While growth is a primarily viscous process, it is helpful to allow non-yielded walls in particular to sustain an elastic stress **σ**_*e*_. We adopt a simple linear relationship between stress and elongational strain with stiffness λ, as shown in more detail below.

**Figure 3:**
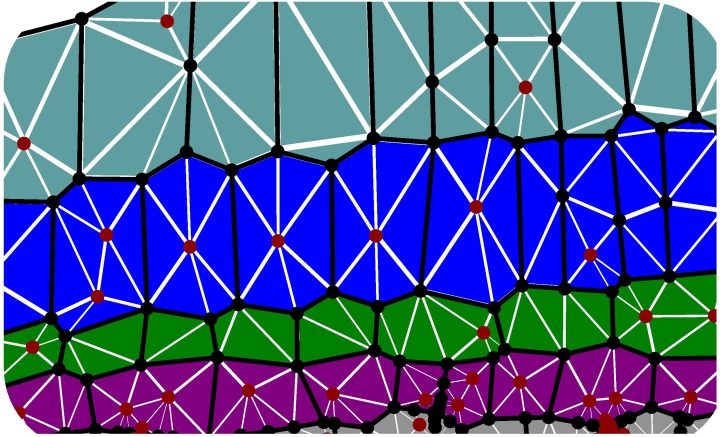
**Triangulated cell walls in the plane of the simulation**. Edges of cells in the plane (axial or cross walls) are shown as heavy black lines; vertices are shown as black or red circles. Note that vertices may lie in the interior of cells (red circles); these are required to avoid using triangles with large aspect ratio in the initial configuration. As the tissue elongates, triangle aspect ratios increase, and either a more highly refined triangular mesh or periodic remeshing is required to accurately simulate organ bending (see Figure 6).

**Figure 4:**
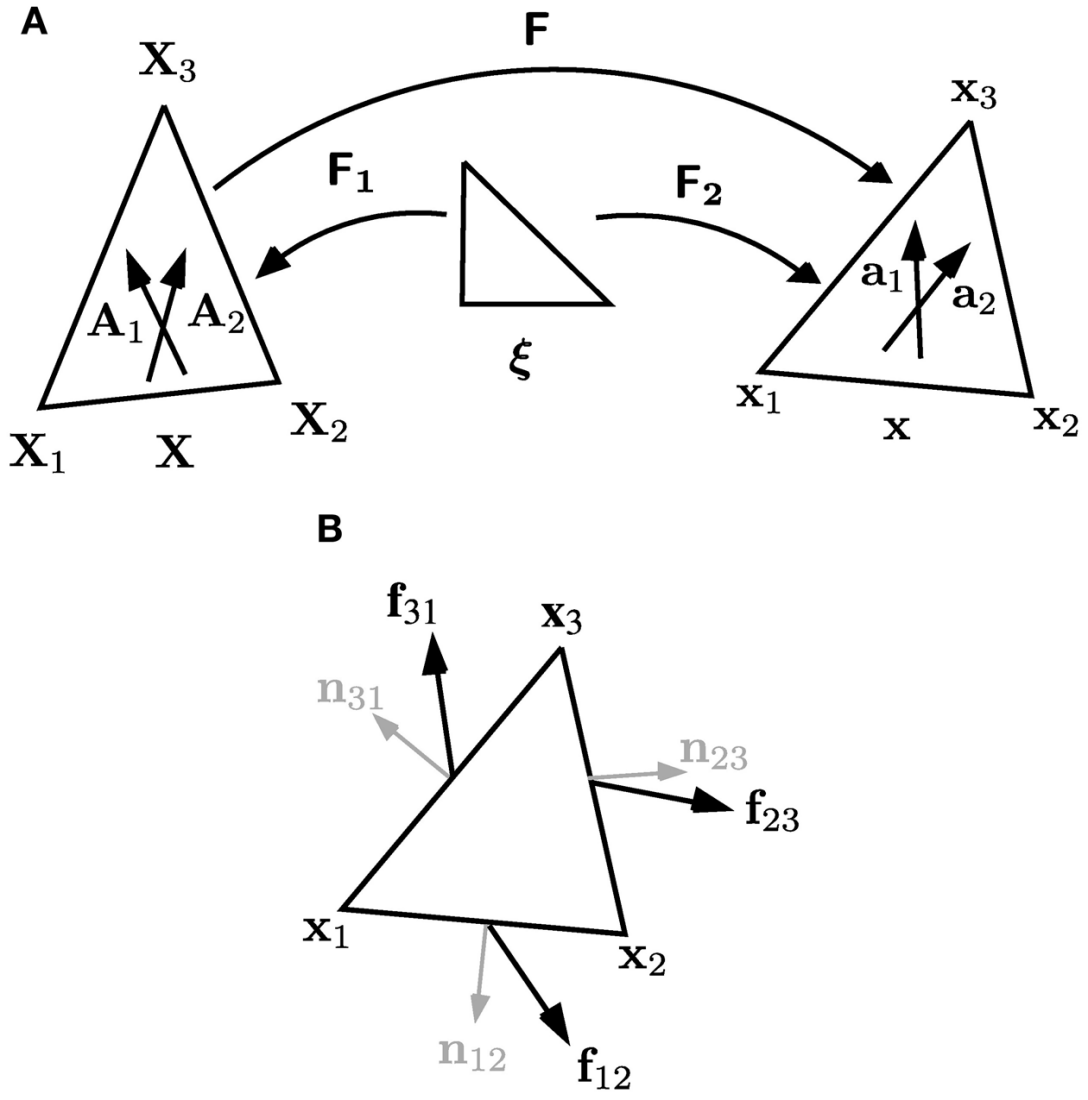
**Coordinate systems and forces for triangular elements lying within walls in the plane of the simulation. (A)** Vertices of a triangular element have positions **X**_1_, **X**_2_ and **X**_3_ in the reference configuration, and **x**_1_, **x**_2_ and **x**_3_ in the current configuration. The gradient of the deformation **F** is calculated using **F** = **F**_2_ **F**^−1^_1_, where **F**_1_ and **F**_2_ are the gradients of the maps, defined by linear interpolation, from the unit triangle to the reference and current configurations, respectively. The elements each have a pair of microfibril directions, **A**_1_ and **A**_2_, in the reference configuration; these are embedded in the material of the cell wall, so are deformed with it to directions **a**_1_ and **a**_2_ in the current configuration. **(B)** Notation used in Equations (16) and (17) for the forces on and normals to the edges of each triangular element.

***2.1.1.3. Cell wall discretization***. Cell walls in the plane of the simulation play an important role in preventing transverse swelling and in determining the rate of axial elongation. For such walls we make the constitutive assumption **σ** = **σ**_*a*_ + **σ**_*y*_. To simulate their properties, each cell wall is sub-divided into triangular elements (see Figure 3). We now describe how to calculate the forces exerted by a particular element on its vertices, without including the element label to simplify our notation. Such calculations are common in implementations of finite element methods—cf. Zienkiewicz and Taylor (2000) or (Bonet and Wood, 2008). Most vertices will be associated with multiple triangular elements (see Figure 3), and so the total force acting on each vertex will be the sum of the contributions from all triangles for which it is a corner. Each element has a pair of fiber directions **A**_1_, **A**_2_ in the reference configuration. These fiber directions map to **a**_1_, **a**_2_ in the current configuration, which are calculated using

(8) $$a_{k}=\frac{\text{F}A_{k}}{\left|\text{F}A_{k}\right|},\;k=1,2,\text{ F}={\text{ F}}_{2}{\text{F}}_{1}^{-1},$$

where **F**_1_ and **F**_2_ are the gradients of the maps from the unit triangle [with vertices at (0, 0), (1, 0), (0, 1)] to the reference and current configurations, respectively (see Figure 4A). These maps are given by linear interpolation, so that position vectors in the two configurations can be written as

(9) $$X(\xi )=\sum_{I=1}^{3}X_{I}N_{I}(\xi ),\;x(\xi )=\sum_{I=1}^{3}x_{I}N_{I}(\xi ),$$

where **ξ** = (ξ_1_, ξ_2_) parameterizes points in the unit triangle, the shape functions *N*_1_, *N*_2_, *N*_3_ are defined by

(10) $$N_{1}=1-\xi_{1}-\xi_{2},\;N_{2}=\xi_{1},\;N_{3}=\xi_{2}$$

and **x**_*I*_(*t*) (*I* = 1, 2, 3), **X**_*I*_(*t*) (*I* = 1, 2, 3) are the positions of the vertices of the triangles in the current and reference configurations (see Figure 4A). The gradients **F**_1_ and **F**_2_ are therefore given by

(11) $$\begin{array}{l} {\left(F_{1}\right)}_{\alpha B}=\frac{\partial X_{\alpha }}{\partial \xi_{B}}=\sum_{I=1}^{3}X_{I\alpha }N_{I,\;B}\left(\xi \right),\\ {\left(F_{2}\right)}_{\alpha B}=\frac{\partial x_{\alpha }}{\partial \xi_{B}}=\sum_{I=1}^{3}x_{I\alpha }N_{I,B}\left(\xi \right), \end{array}$$

where

(12) $$N_{I,\;B}(\xi )\equiv \frac{\partial N_{I}}{\partial \xi_{B}}$$

and *X*_*I*α_ denotes the α component of **X**_*I*_ (α = 1, 2;*B* = 1, 2).

The velocity **v** of a material point is given by

(13) $$v=\sum_{I=\;1}^{3}v_{I}N_{I}(\xi ),$$

where **v**_*I*_(*t*) = ∂**x**_*I*_/∂*t* (the Lagrangian derivative; *I* = 1, 2, 3) is the velocity of the *I*th vertex of the triangular element in which the point sits. The rate of strain tensor Ε appearing in Equation (5)–(7) is then

(14) $$Ε=\frac{1}{2}\left(\;\nabla v+{\left(\nabla v\right)}^{T}\right),$$

where the spatial velocity gradient ∇**v** is given by

(15) $${\left(\nabla v\right)}_{\alpha \beta }=\sum_{I=1}^{3}\sum_{B=1}^{2}v_{I\alpha }N_{I,\;B}(\xi ){\left(F_{2}^{-1}\right)}_{B\beta },$$

We calculate the forces from a triangular element on each of its vertices in a similar manner to the pressure forces, as illustrated in Figure 4B. The forces **f**_12_, **f**_23_, **f**_31_ on each of the edges of the triangle are given by

(16) $$\begin{array}{l} f_{12}=-\sigma \cdot n_{12}|x_{2}-x_{1}|\;f_{23}=-\sigma \cdot n_{23}|x_{3}-x_{2}|\\ f_{31}=-\sigma \cdot n_{31}|x_{1}-x_{3}| \end{array}$$

where **σ** is given by Equation (4) with no elastic component (**σ**_*e*_ ≡ 0); the outward-pointing normals to each of the edges of the triangle are

(17) $$\begin{array}{l} n_{12}=\frac{\text{R}(x_{2}-x_{1})}{\left|x_{2}-x_{1}\right|},\;n_{23}=\frac{\text{R}(x_{3}-x_{2})}{\left|x_{3}-x_{2}\right|},\\ n_{31}=\frac{\text{R}(x_{1}-x_{3})}{\left|x_{1}-x_{3}\right|}. \end{array}$$

Distributing the force on each wall equally to the vertices at either end, the forces **f**_1_, **f**_2_, **f**_3_ associated with stretching of cell walls on the three corners of the element are

(18) $$f_{1}=\frac{1}{2}(f_{12}+f_{31}),\;f_{2}=\frac{1}{2}(f_{23}+f_{12})\;f_{3}=\frac{1}{2}(f_{31}+f_{23}).$$

The contributions from all elements are combined to give to total force from the walls in the plane on each vertex.

Cell walls perpendicular to the plane of the simulation are considered as viscoelastic; whilst the model is similar to that for cell walls in the simulation plane, for simplicity we do not include the effects of microfibril orientation, setting **σ**_*a*_ = 0 in Equation (4). Furthermore, because in our 2D simulation the walls perpendicular to the plane between adjacent vertices can only undergo expansion, we can restrict attention to a single elongational stress component in the wall direction. However, it is helpful in simulations to retain an elastic element in each “cross” wall, as these grow more slowly and are not clearly in a yielded state. For the purposes of mechanical calculations, we treat an edge “viewed from” each of the cells that border it as two distinct wall segments, and reference them by the pair (*m, e*), where *m* is the index of the cell and *e* the index of the edge. For symplastic growth, the two wall segments corresponding to a given edge have the same length and hence strain-rate.

From the constitutive law (Equation 4), the tensile stress resultant in the wall (*m, e*) is assumed to be

(19) $$\sigma_{\left(m,\;e\right)}=2\left(\frac{\tau_{\left(m,e\right)}}{\varepsilon_{\left(m,\;e\right)}^{*}}\left(1-\exp\left(-\frac{\varepsilon_{\left(m,e\right)}^{*}}{\varepsilon_{e}}\right)\right)+\mu_{\left(m,e\right)}\right)\varepsilon_{e}+\lambda_{\left(m,e\right)}\left(\frac{l_{e}}{l_{0}{}_{\left(m,\;e\right)}}-1\right)$$

where ε_*e*_ is the rate of strain of the edge, μ_(*m, e*)_ is the viscosity of the wall, ε^*^_(*m, e*)_ is the yield strain-rate, τ_(*m, e*)_ is the yield stress, *l*_*e*_ is the current length of the edge, *l*_0__(*m, e*)_ is the rest length of the wall and λ_(*m, e*)_ is the spring constant of the wall. The resulting contributions to the forces on the vertices are

(20) $$\pm \sigma_{\left(m,e\right)}\frac{x_{j}-x_{i}}{\left|x_{j}-x_{i}\right|},$$

where the positive and negative signs correspond to the forces on **x**_*i*_ and **x**_*j*_, respectively.

**Figure 5:**
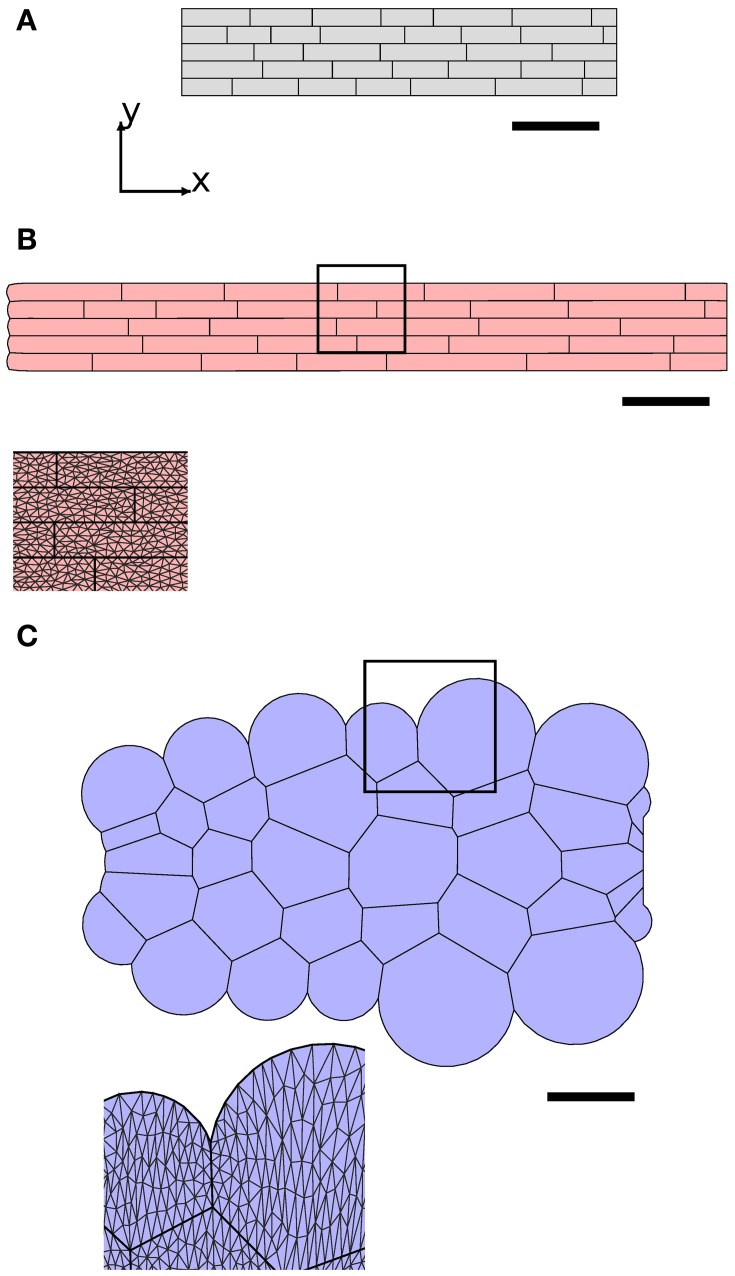
**Viscous cell walls in the plane of the simulation avoid transverse swelling of cells**. The synthetic geometry **(A)** was used to initialize simulations, with results at *t* = 1 hr shown in **(B)** and **(C)**. In **(B)**, with large anisotropic wall viscosity, cells retain their rectangular shape and elongate in the *x*-direction, whereas in **(C)**, with no anisotropic viscosity and only small isotropic wall viscosity (μ_1_ = 0.002 MPa hr, μ_2_ = μ_3_ = 0 MPa hr), cells become rounded. Scale bars indicate 100 μm. Parameters are as listed in Table 1, except that μ_axial_ = 10.0 MPa μm hr in **(C)** to compensate for loss of cell-wall viscosity in the plane of simulation. 7772 triangular elements were used for the cell walls in the plane of the simulation; these elements are visible in the magnified images.

**Figure 6:**
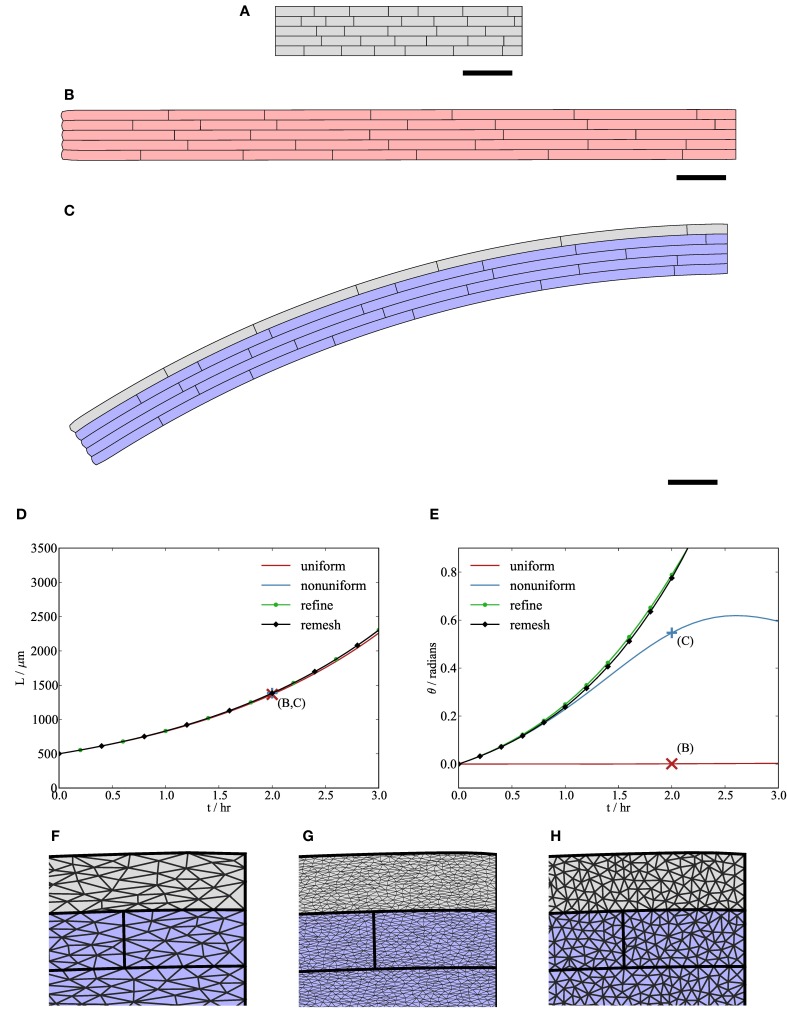
**Variations in cell mechanical properties lead to organ-scale bending. (B,C)** Simulation results at *t* = 2 hr with initial synthetic geometry **(A)**. The gray cells in the uppermost file of the organ in **(C)** have slightly lower isotropic viscosity (μ_1_ = 0.13 MPa hr) than the other cells in **(B)** and **(C)** (μ_1_ = 0.15 MPa hr). **(D,E)** The total length of the midline of the organ *L* and the angle θ made between the tip and the *x*-axis, respectively. Initially, both organs grow roughly exponentially; however, the bending rate may decrease after a while if triangular elements become highly elongated **(F)**, in which case linear interpolation over them gives a poor approximation to the strain rate of the cell walls. This computational artifact can be avoided either by generating a more refined initial mesh (“refine”, **(G)**) or by periodically regenerating the triangulation of the tissue (“remesh”, **(H)**), as shown in **(D)** and **(E)**. Crosses marked **(B,C)** in (**D, E**) correspond to solutions **(B,C)** at *t* = 2 hr. Parameters are as listed in Table 1. Scale bars indicate 100 μm.

**Figure 7:**
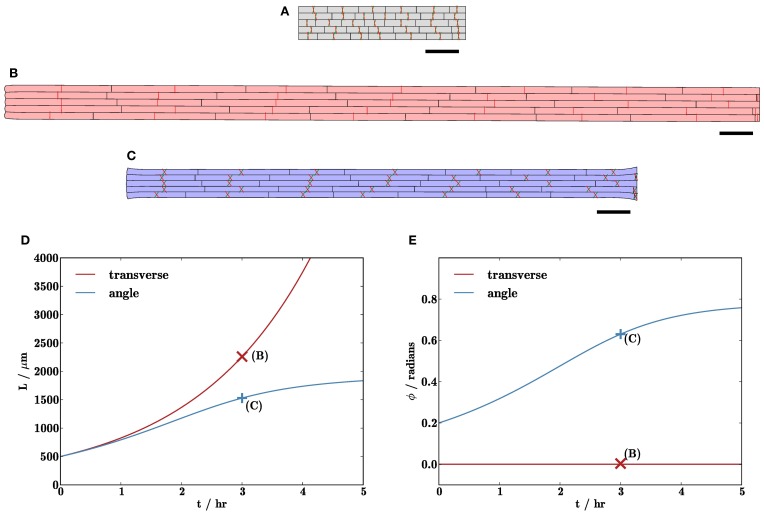
**Cell wall microfibril reorientation can slow organ elongation. (B,C)** Simulation results after 3 hr with initial tissue geometry displayed in **(A)**. In **(B)**, microfibrils (indicated by red and green lines) are initially aligned with the *y*-axis, whilst those in **(C)** are oriented at angles of ± 0.2 to the *y*-axis. **(D)** shows the lengths of the organs, and **(E)** shows the mean angle ϕ between the microfibril orientations and the *y*-axis. The organ in **(B)** grows (roughly) exponentially, whilst the elongation rate of the organ in **(C)** slows as the microfibrils become more closely aligned with the direction of expansion. Triangulation of geometry has 7722 elements; results with refined initial mesh or periodic remeshing (not shown) are very similar to those shown here (<1% relative difference). Markers on **(D,E)** show solutions (**B,C**) at *t* = 3 hr. Parameters are as listed in Table 1. Scale bars indicate 100 μm.

**Figure 8:**
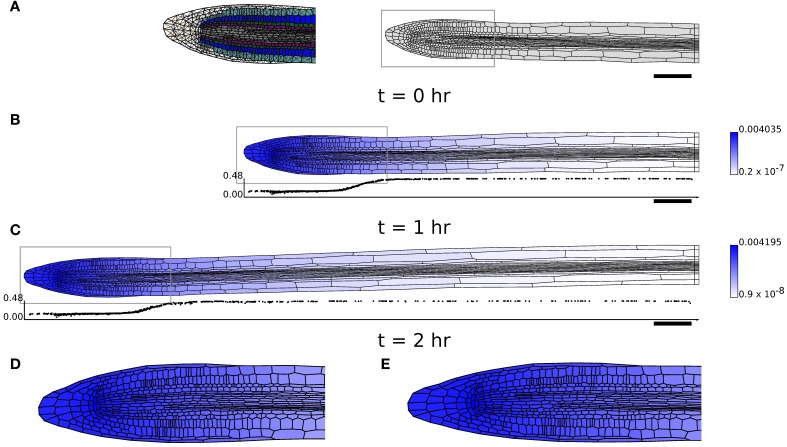
**Simulation of a growing Arabidopsis root apex. (B,C)** Simulation results at *t* = 1 hr and *t* = 2 hr with initial geometry **(A)**. Shading indicates cell growth inhibitor concentration (on a logarithmic scale); graphs below (**B,C**) show the expansion rate of the cells (hr^−1^), plotted at the *x*-position of the cell centroids. **(D,E)** The regions highlighted in gray in **(B,C)** in more detail. Triangulation of geometry has 8937 elements (see **(A)**). Simulation parameters are as in Table 1, except μ_axial_ = 0.05 MPa μm hr, τ^0^_axial_ = 0.1 MPa μm, μ_1_ = 0.3 MPa hr, τ^0^ = 0.1 MPa for (**B,C**) and μ_axial_ = 0.5 MPa μm hr, τ^0^_axial_ = 0.1 MPa μm, μ_1_ = 0.2 MPa hr, τ^0^ = 0.1 MPa for **(D)**.

The non-linear model (Equation 19) reduces to a linear model when τ_(*m, e*)_ = 0; as for the in-plane walls, we use the linear model for simulations on artificial geometries (Figures 5–7), and the non-linear model (τ_(*m, e*)_ > 0) for simulations on realistic root geometries (Figure 8). For simulations on an artificial geometry, cross walls are considered to be viscoelastic (λ_(*m, e*)_ = λ_cross_ = const, μ_(*m, e*)_ = μ_cross_ = const in Equation (19)), but axial walls are treated as purely viscous (λ_(*m, e*)_ = 0, μ_(*m, e*)_ = μ_axial_); for the simulations of Figures 5–7, cell wall properties are assumed to be constant in time, but we permit differences in axial cell wall extensibilities between different cells. In the simulation in Figure 8 below, cross walls have constant and uniform properties (τ_(*m, e*)_ = τ_cross_ and ε^*^_(*m, e*)_ = ε^*^_cross_), axial walls have constant and uniform yield strain rate ε^*^_(*m, e*)_ = ε^*^_axial_, but the axial wall yield stress τ_(*m, e*)_ varies between cells and with time in a manner explained in section 2.1.2 below.

#### 2.1.2. Diffusible growth inhibitor

For simulations in a realistic geometry, it is necessary to specify the mechanical parameters of individual cell walls to capture observed patterns of growth. In order to demonstrate the capabilities of this method, we consider a growth regulator which inhibits growth, with concentration *b*_*m*_ (where *m* runs over the indices of all cells). This is a relatively simplistic model for the regulation of cell mechanical properties by hormones, but it provides a framework that is readily generalized. Chavarría-Krauser et al. (2005) proposed a related model for the regulation of growth in a line of cells, where cytokinin produced in the cell at the tip of the root played similar role to the growth inhibitor here. Muraro et al. (2013) also proposed a model for the regulation of growth in the root involving auxin and cytokinin signaling.

The concentration of the representative growth regulator is assumed constant within each cell and transported between cells only by diffusion through cell walls. The cellular concentrations *b*_*m*_ evolve according to

(21) $$\begin{array}{l} \frac{\text{d}b_{m}}{\text{d}t}=\frac{1}{A_{m}}\sum_{n\in {ℬ}_{m}}\left(P_{b}S_{m,n}(b_{n}-b_{m})\right)-\lambda b_{m}+\alpha_{m},\\ \;m=1,\ldots ,N_{c} \end{array}$$

where ${ℬ}_{m}$ is the set of indices of cells adjacent to the cell with index *m, A*_*m*_ is the area of the cell with index *m, P*_*b*_ is a permeability coefficient, *S*_*m, n*_ is the length of the wall between the two cells with indices *m* and *n*, α_*m*_ is the production rate of substance *b* in cell *m*, λ is the decay rate of substance *b* and *N*_*c*_ is the total number of cells. In our realistic root geometry, each cell has an additional attribute labeling its tissue type. We consider production of *b* to occur only in those cells which are part of the quiescent center (QC). The growth inhibitor concentration serves as a proxy for distance from the QC, and controls growth through modulation of the yield stress of walls in the plane of the simulation, τ_*w*_ (see Equation 7), and the yield stress of axial walls perpendicular to the plane of the simulation, τ_axial_ (see Equation 19). Specifically, we assume that

(22) $$\tau_{w}=\frac{\tau_{w}^{0}}{\left(1+{\left(\frac{k_{b}}{b_{m}}\right)}^{n_{b}}\right)},\;\tau_{axial}=\frac{\tau_{axial}^{0}}{\left(1+{\left(\frac{k_{b}}{b_{m}}\right)}^{n_{b}}\right)}.$$

The half-saturation constant, *k*_*b*_, is chosen to control the position at which rapid cell elongation begins and the Hill constant, *n*_*b*_, controls the sharpness of this transition.

#### 2.1.3. Cell division

In order to further demonstrate the capabilities of this computational approach, we integrated a simple model for cell division within this mechanical framework. Both the timing of cell division (Dissmeyer et al., 2009) and the choice of cell division plane (Sahlin and Jönsson, 2010; Besson and Dumais, 2011; Alim et al., 2012) have been modeled previously. Here we employ a simple model (related to that of Chavarría-Krauser and Schurr, 2004) to specify the division time of cells. Each cell has an associated variable *t*_*m*_, which is initially taken to be a random number drawn from a uniform distribution on [0, 1). This increases with time in all cells at a rate β. A cell will divide provided *t*_*m*_ > 1 and the growth inhibitor concentration is sufficiently large (*b*_*m*_ > *k*_*b*_). Following cell division, *t*_*m*_ for both daughter cells is set to zero. Such a model results in a rate of cell division which is roughly constant in the meristem and zero outside it, as observed by Beemster and Baskin (1998). It also requires cells to wait a period 1/β inbetween successive divisions.

We choose to divide cells perpendicular to the direction of maximum strain rate (Hejnowicz, 1989; Nakielski, 2008). This direction is obtained by calculating the area-weighted average of the rate of strain tensor Ε (Equation 14) over the triangles representing the wall of the cell in the plane of the simulation. This is a symmetric tensor, and the direction of maximum growth is given by the eigenvector associated with the largest eigenvalue. The plane of division is chosen to be that which passes through the geometric center (centroid) of the cell, perpendicular to the direction of maximum growth.

When a cell divides, the polygon representing the walls of the cell perpendicular to the plane of the simulation is divided into two along the plane of division. This requires subdividing two edges of the parent cell into two; a new triangulation is generated for the cells which share these edges and the two daughter cells. This new triangular mesh is obtained from a constrained Delaunay triangulation of the region. This mesh may contain new vertices, and the positions of these in the reference configuration (**X**_*I*_) are calculated by barycentric interpolation. The microfibril directions in the reference configuration, **A**_1, 2_, are constant and uniform within each cell in the simulations of this paper, and the fiber directions for the daughter cells are set to be the same as those of the parent cell.

#### 2.1.4. Computational implementation

The majority of the simulations was implemented in Python, using the OpenAlea (Pradal et al., 2008) simulation environment, which provides data structures for representing vertex-based tissues and routines to manuipulate them. Computationally intensive parts of the simulations (such as solution of mechanical equations) were implemented in C++. Constrained Delaunay triangulations of cell walls in the plane of the simulation were obtained using Triangle (Shewchuk, 1996). All simulations were performed on a standard desktop workstation (16 GiB RAM, 3.4 GHz quad core Intel processor).

An implicit system of differential equations for the vertex positions is given by equating the total forces on each vertex to zero, obtained by adding the pressure forces (Equation 3) from each cell, the forces (Equation 20) from each out-of-plane wall segment, and the in-plane forces (Equation 18) from each triangular element. This system (with c. 12,000 degrees of freedom for the simulations of Figure 8), is solved numerically using the backward Euler method or the differential-algebraic solver (IDA) from the SUNDIALS suite (Hindmarsh et al., 2005). Both schemes use the Newton–Raphson method for the solution of non-linear systems of equations, see e.g., Iserles (2009). The Jacobian matrix is calculated numerically using finite differences, with the known sparsity pattern used to group terms such that multiple columns can be calculated simultaneously. The direct sparse solver UMFPACK (Davis and Duff, 1999) is used for linear algebra. IDA is used for the simulations of Figures 5–7, as it uses an adaptive stepsize method to control the error in the solution. However, we use the backward Euler method for the simulations of Figure 8, as this proves more robust for simulations on complicated geometries.

In simulations on a realistic geometry, the equations for the growth inhibitor (Equation 21) form an additional system of ordinary differential equations which are coupled to the mechanical model. For simplicity of implementation, we use a split-timestep method, in which we simulate each of the chemical and mechanical components of the model in turn over a fixed timestep. The calculated concentrations of growth inhibitor in each cell are then considered to be constant for the simulation of the mechanical parts of the model over one timestep. Cell division is considered at the end of each timestep.

#### 2.1.5. Synthetic geometry

In order to investigate basic mechanical features of the model, a rectangular “root” was generated, with *N*_*y*_ = 5 files of cells of length *L*_*x, tot*_ = 2000 μm in the *x*-direction and height *L*_*y*_ = 20 μm in the *y*-direction. This height is comparable to the diameter of an Arabidopsis cortical cell (Dolan et al., 1993), although it should be noted that the other cell files in the real root are of smaller diameters. Each file of cells was further subdivided in the *x*-direction (starting at the wall with smallest *x*-coordinate, corresponding to the rootward end) with each cell length drawn randomly from the uniform distribution on [*L*_*x*_, 2*L*_*x*_], where *L*_*x*_ = 50 μm.

#### 2.1.6. Realistic geometry

Longitudinal sections of Arabidopsis roots, with cell walls highlighted by propidium iodide staining, were obtained using confocal microscopy. These images were traced by hand using a vector graphics package (Inkscape). The OpenAlea environment (Pradal et al., 2008) provides routines to import tissues from Scalable Vector Graphics files. The root was aligned such that the long axis of the root was parallel to the *x*-axis. Edges were classified as axial or cross according to the angle between the edge and the *x*-axis (those edges within π/4 being axial and the others cross).

### 2.2. Parameter estimates

Turgor pressures within Arabidopis have been measured to be of the order of 0.3 MPa (e.g., see Javot et al., 2003). The remaining parameters in the model are difficult to measure experimentally; the viscosity of an elongating cell wall is an emergent property arising from the dynamic breaking of hemicellulose crosslinks and the viscosity of the pectin matrix. However, cell elongation rates within the root have been measured by Peters and Baskin (2006) to be about 0.1 hr^−1^ near the QC, and 0.5 hr^−1^ in the center of the elongation zone, and this guides our choices of parameter values, as described below. In the absence of independent information, the main criterion for these choices was that they gave biologically sensible results. Note that the growth profile of roots has been measured to vary significantly between different seedlings (Walter et al., 2002); a number of other authors have also measured growth profiles in roots using modern techniques (Walter et al., 2003; van der Weele et al., 2003; Basu et al., 2007; Chavarría-Krauser et al., 2008).

For an isolated rectangular cell of length *L*(*t*) and uniform width *H*, with fibers oriented perpendicular to its primary axis, we have from Equation (7) and (19), neglecting yield, that

(23) $$Hp=\left(2\mu_{1}H+4\mu_{axial}\right)\varepsilon ,\;\varepsilon =\frac{1}{L}\frac{\text{d}L}{\text{d}t},$$

showing how in-plane and side walls together regulate elongation. Taking

(24) $$\mu_{1}\approx \frac{p}{4\varepsilon },\;\mu_{axial}\approx \frac{Hp}{8\varepsilon },$$

with ε = 0.5 hr^−1^, the axial cellular elongation rate will be of the correct order, and the walls parallel and perpendicular to the plane of the simulation will make similar contributions to the forces resisting elongation. Yield strain rates (ε^*^, ε^*^_axial_) are chosen such that all axial walls and cell walls in the plane of the simulation (in the meristem and elongation zones) are in a yielded state. Parameters for cross walls and the anisotropic components of the viscosity in the plane of the simulation (μ_2_ and μ_3_) were chosen to give minimal cell growth in the lateral direction. For the growth inhibitor, we took the permeability coefficient to be *P*_*b*_ = 2 × 10^3^ μm hr^−1^ (following the estimates for auxin from Kramer, 2004); the decay and production rates were chosen to give a plausible elongation rate profile (Walter et al., 2002; van der Weele et al., 2003; Peters and Baskin, 2006; Chavarría-Krauser et al., 2008) along the root axis. A full list of parameter values is given in Table 1.

**Table 1:** **Table of default parameter values**.

**Symbol**	**Description**	**Default value**	**Units**
*p*_*m*_	Pressure	0.3	MPa
μ_cross_	Cross wall viscosity	0.05	MPa μm hr
λ_cross_	Cross wall spring constant	100	MPa μm
τ_cross_	Cross wall yield stress	0	MPa μm
ε^*^_cross_	Cross wall yield strain rate	0.05	hr^−1^
μ_axial_	Axial wall viscosity	1.5	MPa μm hr
τ_axial_	Axial wall yield stress	0	MPa μm
ε^*^_axial_	Axial wall yield strain rate	0.05	hr^−1^
μ_1_	Wall isotropic viscosity	0.15	MPa hr
μ_2_	Wall anisotropic (extensional) viscosity	20.0	MPa hr
μ_3_	Wall anisotropic (shear) viscosity	0	MPa hr
ε	Wall yield strain rate	0.05	hr^−1^
τ_*w*_	Wall yield stress	0	MPa
*P*_*b*_	Wall permeability for growth inhibitor	2 × 10^3^	μm hr^−1^
λ_*b*_	Growth inhibitor decay rate	16	hr^−1^
α_*b*_	Growth inhibitor production rate in QC cells	2	nM hr^−1^
*k*_*b*_	Half saturation constant for growth inhibitor	1 × 10^−5^	nM
*n*_*b*_	Hill coefficient for growth inhibitor action	2	n/a
τ^0^_axial_	Axial wall yield stress	0.1	MPa μm
τ^0^_*w*_	Basal wall yield stress	0.1	MPa
β	Cell division rate	0.1	hr^−1^
*N*_*y*_	Number of cell files in synthetic geometry	5	n/a
*L*_*y*_	Radial size of cell files	20	μm
*L*_*x*_	Minimum (and half maximum) axial cell length	50	μm

## 3. Results

We initially (Figures 5–7) consider simulations on a synthetic geometry, with constant mechanical properties for individual cells prescribed at the start of the simulation and not including growth regulation by diffusible substances. We use models with linear viscous terms (τ = 0, τ_*w*_ = 0), allowing us to explore the properties of the mechanical model in more detail. Later (Figure 8), we will consider simulations on a realistic geometry, and include growth controlled by a diffusible growth inhibitor and non-linear viscous terms in order to represent the yielding behavior of cell walls. Unless otherwise noted, all parameters are as listed in Table 1.

Figure 5 shows the importance of the mechanical properties of cell walls in the plane of the simulation in promoting elongation. We consider two simulations with identical synthetic geometries as their initial configuration (shown in Figure 5A). In Figure 5B, cell walls in the plane of the simulation have significant viscosity, both isotropic (μ_1_) and parallel to the microfibrils (μ_2_), which initially lie in the (transverse) *y*-direction. We find that the organ elongates preferentially in the (axial) *x*-direction, with cells maintaining a rectangular shape, demonstrating how fiber reinforcement allows anisotropic expansion. Note that this is a consequence of our choice of substantial viscosity μ_2_ in the microfibril direction. In Figure 5C, the viscosity of the cell walls in the plane is reduced to a very small value (μ_1_ = 0.002 MPa hr), but remains non-zero in order to regularize the numerical solution, and the anisotropic components of the viscosity (μ_2_, μ_3_) are set to zero. We increase the viscosity of axial walls (μ_axial_ = 10 MPa μm hr) in order to compensate for the loss of viscosity in the plane of the simulation. The cells bulge and become rounded, as there are no forces acting to hold together their axial sides. This behavior is similar to the transverse expansion of cortical cells arising when the elongation of endodermal cells is inhibited through blocking their response to gibberellic acid (Ubeda-Tomas et al., 2008).

Figure 6 shows how cell-scale variations in mechanical properties can lead to organ-scale behavior. All the cells in the “uniform” case, shown in Figure 6B, have the same isotropic viscosity in the plane of the simulation (μ_1_ = 0.15 MPa hr), along with significant viscosity parallel to the microfibrils (μ_2_ = 20.0 MPa hr), which causes the organ to elongate in the *x*-direction. The “non-uniform” case, shown in Figure 6C, is identical to the uniform case, except that cells in the uppermost file (gray) have lower isotropic viscosity (μ_1_ = 0.13 MPa hr). In both cases, the initial configuration is as in Figure 6A. The graph of organ length against time in Figure 6D shows that the difference in the length of the midline of the organ between the uniform and non-uniform cases is small. However, the angle θ made between the left-hand end of the growing organ and the *x*-axis, shown in Figure 6E, indicates a clear bending response (at *t* = 2 hr) in the non-uniform case, as shown in Figure 6C. In the absence of adhesion to their neighbors, cells with lower viscosity would extend more rapidly; as the cells are tightly adherent to each other, the organ bends. The tip angle would be expected to be an increasing (and accelerating) function of time, but simulation results in Figure 6E do not always show this. This is because, for an organ undergoing bending, the finite element discretization provides a less accurate approximation to the strain rate tensor in the plane of the simulation as the aspect ratio of the triangular elements increases (during elongation of the organ), as shown in Figure 6F. In Figures 6G,H we illustrate two approaches to avoid this effect. In the first one (“refine”), we use a finer spatial discretization (77,653 triangular elements, compared with 7722 in the “uniform” and “non-uniform” cases). With the finer mesh, the tip angle is an accelerating function of time, but the simulation is considerably slower (c. 60 min compared with c. 3 min for the non-uniform case). In the second approach (“remesh”), we regenerate a new constrained Delaunay triangulation of the geometry every 5 timesteps (0.5 hr). This introduces new vertices within the wall of each cell; the positions of these vertices in the reference configuration (**X**) are calculated by barycentric interpolation. The tip angle in this case is in agreement with the results from simulations on the finer mesh, but this method is computationally more efficient (c. 8 min). In summary, care must be taken to avoid erroneous predictions associated with distortion of elements, but practical steps can be taken that do not make computation time excessive.

Figure 7 illustrates the effect upon organ growth of anisotropic viscosity caused by oriented cellulose microfibrils. In particular, it shows how reorientation of microfibrils can slow organ elongation (cf. the multinet model of Green, 1960). In Figure 7B, the microfibrils are initially oriented transversely (parallel to the *y*-axis), whereas in Figure 7C, the microfibrils are initially oriented in a pair of directions making angles ± 0.2 radians to the *y*-axis. Again, the initial configuration is as in Figure 7A. As can be seen from Figures 7D,E, the organ with microfibrils initially parallel to the *y*-axis extends exponentially with time, and there is little reorientation of the microfibrils. In simulations with the microfibrils starting at a small angle to the *y*-axis, initially they do not increase the resistance to elongation significantly, as can be observed in Figure 7D. However, as the organ extends, the angle ϕ made by the microfibrils with the *y*-axis increases (as they are embedded in the material of the wall), as shown in Figure 7E. This increases their contribution to the viscosity in the direction of elongation, thereby slowing the growth of the organ. (The situation in Figure 7B, where microfibrils are perpendicular to the direction of elongation, is not stable to small numerical perturbations; slight asymmetries may cause the microfibril orientations to rotate.) This demonstrates how fiber reorientation alone is sufficient to suppress tissue growth, supporting predictions for individual cells by Dyson and Jensen (2010). Note that, in an actual root, we expect fiber angles to be spatially non-uniform, being aligned more closely with the axis of elongation as we move further away from the root apex.

Figure 8 shows that the computational model can be used to simulate growth on the scale of the root apex of an Arabidopsis seedling. In the present model, we simulate growth by cell expansion alone, ignoring cell division. The geometry used (Figure 8A) consists of the whole root tip and about half of the rapidly growing region (elongation zone), but not the region further away from the tip within which growth slows (the “growth terminating zone”) (Verbelen et al., 2006). In Figure 8, levels of the diffusible growth inhibitor are high near the QC, but decrease along the main axis of the root. This modifies the yield stress of the cell walls [according to Equation (22)], and leads to cells undergoing a rapid transition between slowly growing and rapidly elongating states, as is found in experimental measurements of growing roots (Chavarría-Krauser, 2006; Peters and Baskin, 2006). (The simulations of Figure 8 have 6265 triangles and 12,860 degrees of freedom; the simulation (Figures 8A–C) for 2 hr took less than 2 min.) The local cellular pattern near the root apex at *t* = 1 hr and *t* = 2 hr is shown in Figures 8D,E. Cell divisions can be observed in a number of cells, showing the capability of the model. However, it is clear that additional information about the regulation of growth and division in the region near the QC is necessary for the model to be used to study this region in detail.

**Figure 9:**
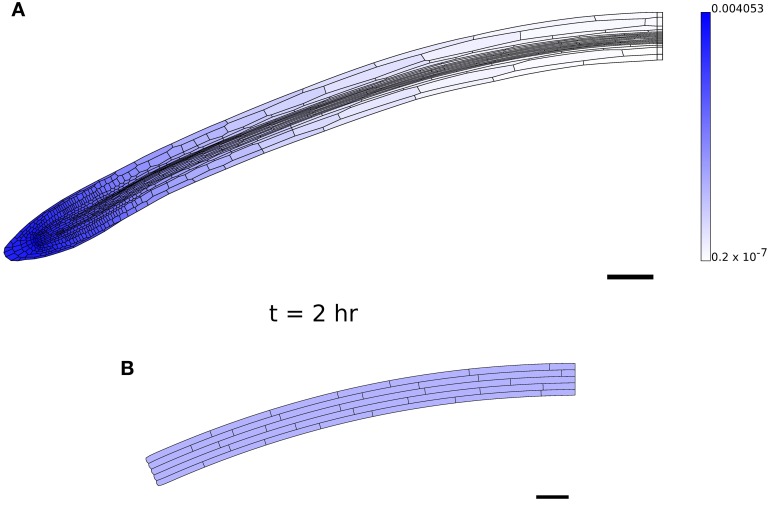
**Bending caused by small asymmetries. (A)** Simulation results at *t* = 2 hr with greater axial wall viscosity μ_axial_ than in Figure 8; bending is a consequence of cells on the lower side of the root being narrower than those on the upper side. **(B)** The result of a simulation on the artificial geometry of Figure 6A at *t* = 2 hr, where the lowest file of cells is slightly thinner (18 μm) than the other files (20 μm); cf. Figure 6C. Simulation parameters are as in Table 1, except μ_axial_ = 0.5 MPa μm hr, τ^0^_axial_ = 0.1 MPa μm, μ_1_ = 0.2 MPa hr, τ^0^ = 0.1 MPa for **(A)**.

In order to obtain simulations which qualitatively agree with observations of growing roots (Figures 8A–C), it proved necessary to modify the simulation parameters, setting μ_axial_ = 0.05 MPa μm hr, τ^0^ = 0.1 MPa, τ^0^_axial_ = 0.1 MPa μm, μ_1_ = 0.3 MPa hr. These changes were made to reduce the importance of the walls perpendicular to the plane of the simulation. However, with greater axial wall viscosity (μ_axial_ = 0.5 MPa μm hr; the isotropic viscosity μ_1_ was decreased to 0.2 MPa hr to increase the overall expansion rate of the organ), it was found that the organ undergoes substantial bending, as shown in Figure 9A. This is a result of the cells on the lower side of the geometry being slightly narrower than those on the upper side; such differences are pronounced in 2D as cell widths depend upon the plane used to generate the 2D section. This effect is illustrated in Figure 9B; this simulation is identical to Figure 6B, except that the bottom file of cells is made to be slightly narrower (18 μm compared with 20 μm), and this results in the organ bending. Despite the limitations of the simulation, this result illustrates the sensitivity of the overall organ morphology to the shape of its constituent cells when one takes full account of the mechanical and kinematic constraints of symplastic growth.

## 4. Discussion

We have presented a 2D vertex-element model for growing plant tissues that allows us to connect detailed mechanical properties of individual cell walls to the shape and growth rate of a whole organ. We showed for example that consideration of cell walls in the plane of the simulation is necessary when cells have a large aspect ratio, as is the case for cells in the elongation zone of *A. thaliana*, otherwise cells bulge and become rounded (Figure 5C). Note that angular springs could also be used to prevent this bulging, and are likely to be less computationally expensive; alternatively, beam elements could be used (Dupuy et al., 2010). However, our model directly includes the wall mechanical property that is believed to be responsible for the anisotropy of growth (Baskin, 2005). The constitutive law used for cell walls in the plane of the simulation incorporates anisotropy generated by cellulose microfibrils, and the yielding of cell walls is modeled using a non-linear viscous law, derived from a model of crosslink turnover (Dyson et al., 2012). While we have not here presented a complete parametric study, our model can nevertheless be used to assess the effect of enzymes that target different components of the cell wall on organ-level morphologies.

Simulations on a synthetic geometry showed how variations in cell scale properties could lead to organ-scale behaviors. These simulations also indicated the difficulty in simulating highly elongated tissues, as the elements in the plane of the simulation also become elongated and so less able to approximate the strain rate in the tissue. We demonstrated a solution to this computational difficulty through periodic remeshing of the tissue (Figure 6E). Our results highlight the importance of careful assessment of numerical errors that can easily arise in simulations of this complexity.

The effects of cellulose microfibrils on plant cell growth were investigated. Microfibrils rotate as the organ elongates, reducing the angle between their direction and the midline of the organ (Anderson et al., 2010). This increases the effective viscosity of the organ along the midline, thereby reducing its growth rate (Figure 7). Such observations support the hypothesis that microfibril reorientation may play a role alongside stiffening of the pectin matrix in determining when cells stop expanding as they leave the elongation zone.

The mechanical model was then applied to simulate a growing root apex (Figure 8), using a geometry acquired from confocal imaging. The yield stresses of cell walls were regulated by a diffusible growth inhibitor. Such a model is intentionally artificial. It is thought that a number of different hormones regulate growth in Arabadopsis roots (Ubeda-Tomas et al., 2012). Whilst the details of some hormone response networks have become sufficiently clear to permit the development of mathematical models (Middleton et al., 2010, 2012b; Dupeux et al., 2011), there are gaps in our understanding of how the levels of these hormones are controlled through synthesis and transport, and the downstream effect of these hormones upon cell wall biochemistry needs further investigation (Sanchez-Rodríguez et al., 2010). There is also only partial information about the effects of the numerous cell wall remodeling enzymes on cell wall mechanical properties, and we did not include spatial variation in the initial microfibril orientations. Moreover, our model does not at present include a number of processes, including the shedding of lateral root cap cells and forces imposed by the external medium, which are important for the maintenance of the structure and shape of the root apex. As the understanding of these effects becomes more well developed, these can be readily included in the current framework.

For simulations of the whole root apex, simulation parameters had to be chosen carefully to avoid bending because of small asymmetries in the geometry (Figures 8C,D). These asymmetries may be partly a consequence of the 2D representation of a 3D tissue, and also because of inaccuracies in the acquisition of the tissue geometry. By stiffening epidermal tissues and softening inner tissues (simulations not shown), we find that the tendency to grow straight is promoted. This may be a benefit of residual stress patterns in elongating organs, or it may compensate for the fact that a 2D projection of a 2D root underestimates the proportion of cells in peripheral tissues.

For roots to grow straight, in the absence of other external stimuli, may require additional mechanisms to coordinate growth, for instance sensing the stress or strain within individual cell walls. Alternatively, this bending may benefit the plant by encouraging the root to explore its local environment. These questions may need 3D models to be properly resolved. Nevertheless, it is notable that our method can be implemented to produce uniform elongation of a structure that has internal asymmetries in its architecture. It will be of significant interest to understand in more detail the interplay between geometric asymmetries and gradients in mechanical properties in processes such as gravitropic bending, extending existing models for root gravitropism (Barlow et al., 1989; Stočkus and Moore, 1996; Chavarría-Krauser, 2006; Moulia and Fournier, 2009) to include more cell-scale detail. Our results show that small differences in the mechanical properties in the outer tissue layers can lead to substantial curvature generation.

This vertex-element framework can be extended to include a number of biological processes which are important in regulating growth. Water transport between individual cells is regulated by the osmotic potential of the cells and the permeability of the cellular membranes. The latter can be controlled by aquaporins; certain aquaporins were observed to be specifically expressed in certain regions during the development of lateral root primordia (Péret et al., 2012), and a mathematical model which lumped tissues into different compartments elucidated the role of this process in controlling lateral root emergence. The current framework may be readily extended to incorporate non-uniform turgor pressure and water transport between cells, along with water transport in the apoplast and the supply of water to the xylem and from the phloem (Steudle and Peterson, 1998; Passioura and Boyer, 2003; Wiegers et al., 2009). The more detailed representation presented here will be of use in interpreting the importance of cell-scale spatial and temporal variations in aquaporin expression during lateral root emergence.

We have shown that, even in 2D, it is necessary to simulate both the edges and faces of individual cells in order to predict realistic organ-level growth. In extending the present model to 3D, there are a number of key difficulties which need to be overcome. Acquiring accurate 3D geometries for a whole root apex is currently beyond the capabilities of confocal imaging, although much progress has been made for other organs such as the shoot apical meristem (Fernandez et al., 2010). As the orientation of cell walls with respect to the direction of growth is critical, these geometric representations need to be of a very high quality. Finite-element discretization of cell walls in 3D is also more complicated, particularly if the cell walls are represented by shell elements: although many finite element packages implement this, cell division proves to be difficult in such a context. Such considerations illustrate the many challenges that remain in further developing such approaches and reinforce the current value of 2D formulations, both in their own right and in laying crucial foundations for further 3D studies.

### Conflict of interest statement

The authors declare that the research was conducted in the absence of any commercial or financial relationships that could be construed as a potential conflict of interest.
